# Clinical utility and costs of inpatient hereditary thrombophilia testing following acute VTE: A 5-Year retrospective study

**DOI:** 10.1007/s11239-025-03183-2

**Published:** 2025-10-05

**Authors:** Eliakim Munda, Ruben Rhoades

**Affiliations:** Division of Hematology, Department of Medicine, Cardeza Foundation for Hematologic Research, Thomas Jefferson University, Philadelphia, PA USA

**Keywords:** Hereditary thrombophilia, Venous thromboembolism, High-value care, Costs

## Abstract

Hereditary thrombophilia testing is frequently ordered after venous thromboembolism (VTE), despite little evidence of clinical utility and most guidelines cautioning against testing. We conducted a retrospective, observational study of inpatient hereditary thrombophilia testing ordered during a hospital admission for acute VTE between 2019 and 2024. We aimed to characterize patterns of testing results, and costs, and to evaluate whether younger patients and those with unprovoked VTE were more likely to test positive for hereditary thrombophilia. A total of 835 hereditary thrombophilia tests – including those for factor V Leiden, prothrombin_G20210A_, deficiencies of protein S, protein C, and antithrombin, hyperhomocysteinemia, and plasminogen activator inhibitor-1 excess – were ordered in 220 patients. Overall, 19.6% of results were abnormal, and 45.0% of patients had at least one abnormal result. There was no difference in the rate of positive results among patients with provoked vs. unprovoked VTE (30.7% vs. 34.5%, *p* = .554) nor patients < 50 vs. ≥ 50 years of age (33.1% vs. 32.4%, *p* = .912). Only 4/99 (4.0%) patients with an abnormal result had their clinical management clearly changed due to the result. The tests totaled $385,161 USD in institutional charges and $26,029 USD in Medicare fees. Inpatient hereditary thrombophilia testing during admission for acute VTE is low yield, with frequent abnormal results, many of which likely represented false positives, and minimal impact on clinical management with high costs.

**Graphical abstract:**

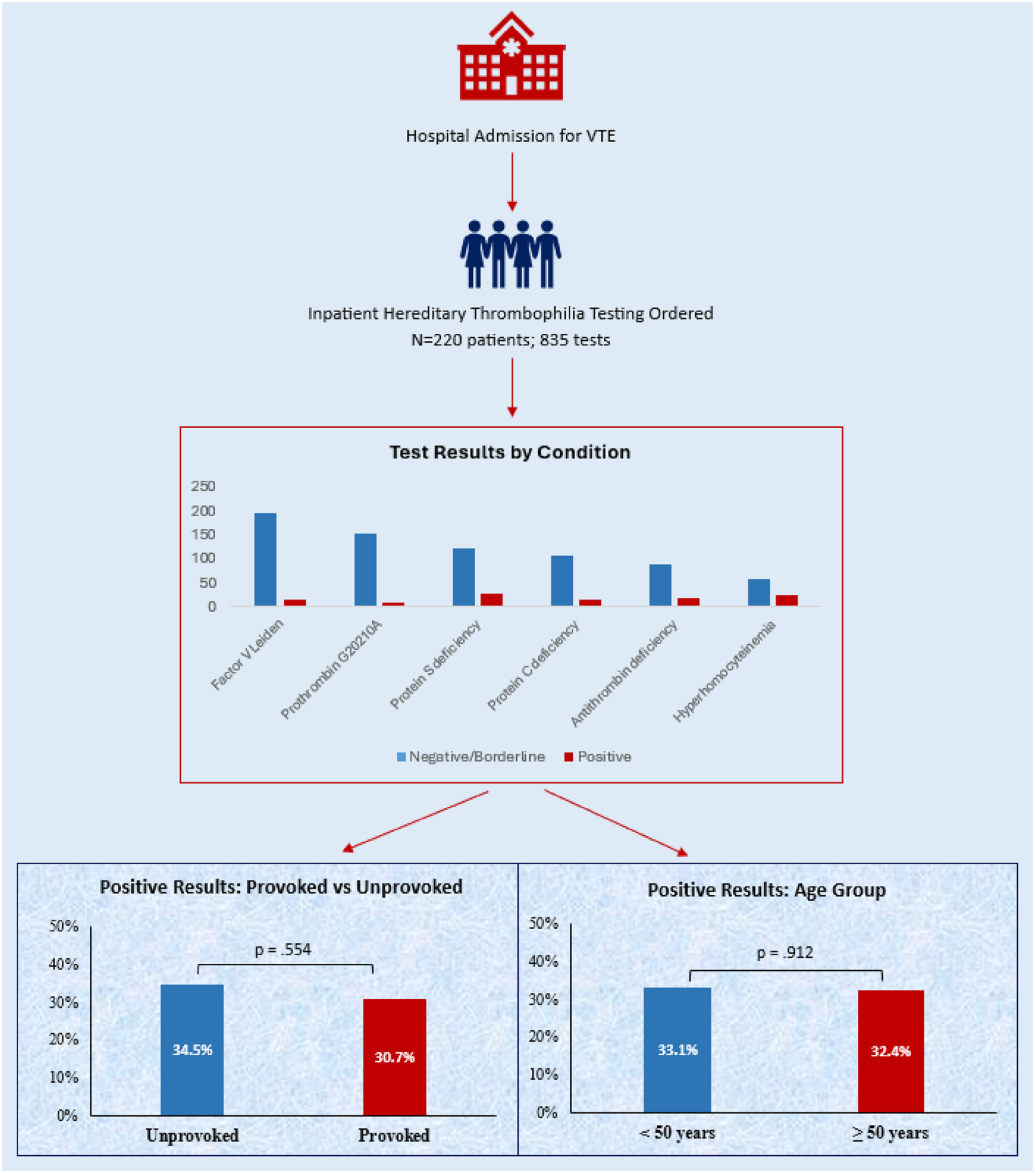

## Key points

- Thrombophilia testing following acute venous thromboembolism was commonly performed at an academic medical center with high associated costs, and nearly 20% of tests were abnormal.
- Many results were below the limits of normal for the assay, but higher than expected in congenital deficiency, likely indicating false positives.
- Young patients and those with unprovoked venous thromboembolism were not more likely to have positive test results.
- Clinical management rarely changed in response to a positive result, indicating low value of testing in the inpatient setting.
- Prospective studies with long-term outpatient follow-up would help determine the optimal timing of thrombophilia testing.

## Introduction

Hereditary thrombophilias are genetic disorders that increase the risk of venous thromboembolism (VTE) [1–4]. Laboratory testing for hereditary thrombophilia is thus a common approach in clinical practice to determine predisposing factors to VTE. However, studies indicate that this screening provides limited utility for predicting the risk of recurrent thrombosis in patients experiencing a first episode of VTE [5–8]. In patients being treated with anticoagulation, the presence of a thrombophilia does not predict a greater recurrence risk [9]. Further, antigen and activity assays for these conditions are susceptible to false positive results immediately following an acute VTE episode, due to factors such as anticoagulation therapy, protein consumption, acute phase reactant changes, and abnormal liver function [10–12]. Given the above, retrospective studies have found that management decisions are rarely influenced by testing [12–14] and positive tests are typically not repeated for confirmation [15]. Finally, tests for hereditary thrombophilias are expensive, with current estimates for a panel of tests costing more than $300 USD [15–17].

Given the limitations and costs, most guidelines have recommended against the use of routine thrombophilia testing, especially when results are unlikely to change a patient’s management [18–21]. The American Society of Hematology (ASH) previously recommended against thrombophilia testing after a VTE provoked by a major transient risk factor [20], however more recent ASH guidelines do conditionally recommend testing in select patients with VTE in whom results may dictate length of anticoagulation, including those with hormone-associated VTE or minor transient provoking risk factors and those with unusual site thrombosis being considered for time-limited anticoagulation [22]. However, these guidelines do not provide guidance on when testing should be ordered, and the risk of inappropriately recommending indefinite anticoagulation is increased when tests are ordered in settings in which false positive results are more likely.

We sought to address this gap in guidelines by studying the pattern and cost of hereditary thrombophilia testing following an acute VTE and how positive results were managed. We hypothesized that in the acute setting, test results would rarely be used to make clinical decisions.

## Methods

### Study population and selection criteria

We retrospectively analyzed all inpatient hereditary thrombophilia tests ordered at Thomas Jefferson University Hospitals (TJUH) – including its three primary flagship hospitals in Philadelphia, PA – an academic tertiary care center, between January 1, 2019 and December 31, 2023. Inclusion criteria comprised patients who underwent inpatient hereditary thrombophilia testing during an admission for acute VTE. Patients who had concurrent VTE and arterial thrombosis/ischemia were also eligible. Types of VTE included deep vein thrombosis (DVT) of the limbs, pulmonary embolism (PE), cerebral venous sinus thrombosis (CVST), splanchnic vein thrombosis (SVT), as well as other rarer sites of VTE such as the inferior vena cava and gonadal veins. Patients who had thrombophilia workups performed for any non-VTE indications, or whose testing was done while inpatient but for a remote VTE, were excluded from the study. Tests for the following conditions were included in the study: Factor V Leiden (FVL; including activated protein C resistance and genetic testing), prothrombin G20210A variant (PT_G20210A_), protein S deficiency (free, total, and functional protein S), protein C deficiency (antigen and function), antithrombin deficiency, hyperhomocysteinemia (homocysteine and *MTHFR* mutation testing), and plasminogen activator inhibitor-1 (PAI-1) excess. The study was approved by the Thomas Jefferson University Institutional Review Board.

### Data collection

We generated a list of all instances of inpatient orders of hereditary thrombophilia tests during the study period, then manually reviewed all charts in our institution’s electronic health record (Epic Systems©) for eligibility, demographics, rationale for testing, provoking risk factors, lab results, and documented management. The International Society on Thrombosis and Haemostasis’ (ISTH) guidance on provoking risk factors [23] was utilized to determine whether a VTE was provoked by a transient risk factor. Additionally, we considered longer-term risk factors such as active cancer or anatomic anomalies (May-Thurner syndrome or venous thoracic outlet syndrome) and transient risk factors not included by the ISTH, such as COVID-19 infection within the preceding 30 days and other conditions associated with splanchnic vein thrombosis (SVT) [24]. We reviewed all available documentation, including after the index hospital admission, to determine if any change in patients’ management was made citing a positive thrombophilia test result. Management changes were considered *definite* if, following a positive result becoming available, a treating clinician prescribed an anticoagulant after having previously not done so, changed the anticoagulant or dose, or extended the treatment duration. We also reviewed occurrences of repeat outpatient orders after initial abnormal results on antigen or functional assays. Last, we queried both TJUH’s chargemaster to determine charges associated with each test and the Centers for Medicare & Medicaid Services’ (CMS) Clinical Laboratory Fee Schedule to obtain payment rates.

For interpreting certain lab results Regarding result interpretation, certain tests – including antithrombin activity, protein C antigen and function, and total protein S – have a lower limit of normal higher than is typically seen in autosomal dominant hereditary deficiencies (in which antigen or activity is typically near or below 50%). We considered values that fell below these labs’ normal range but > 60% to be *borderline positive* to reflect that these were likely not representative of hereditary deficiency, and those < 60% were considered *positive*. Positive genetic assays were characterized as heterozygous, homozygous, or compound heterozygous.

### Statistical analysis

We de-identified and analyzed data using IBM SPSS version 29. Descriptive statistics were summarized according to tests and individual patients. Continuous variables were analyzed using independent sample t tests, and categorical variables Pearson χ^2^ and Fisher’s Exact Test.

## Results

### Patient characteristics

The study included 220 patients, among which 61.8% were female and the median age was 48 years (interquartile range 33–62). 195 (88.6%) patients had only VTE (whether single or multiple sites) and 25 (11.9%) had concurrent VTE and arterial thrombotic or ischemic events. Among patients with single-site VTE, the most common events were PE (27.3%), CVST (21.4%), DVT (15.0%), and SVT (8.2%). 31 (14.1%) patients had VTE at multiple sites, including 25 (11.4%) with concurrent DVT and PE. VTE was considered provoked in 101 (45.9%) patients; 11 (5.0%) patients had at least 2 provoking risk factors. Among patients with provoked VTE, the most common provoking factors were pregnancy/combined oral contraceptive (COC) pills (35.6%), antecedent hospitalization (15.8%), major surgery (13.9%), trauma or prolonged immobility (8.9%), and liver or gastrointestinal disease (7.9%) in the case of SVT. Details on the types of thrombosis and risk factors are shown in Table 1.

**Table 1:** Clinical presentation and risk factors

Presentation	*N* (%)
**Isolated VTE**	**164 (74.5%)**
PE	60 (27.3%)
CVST	47 (21.4%)
DVT	33 (15.0%)
Lower limb	26 (11.8%)
Upper limb	7 (3.2%)
Splanchnic vein thrombosis	18 (8.2%)
Other VTE†	6 (2.7%)
**VTE at Multiple Sites**	**31 (14.1%)**
DVT + PE	25 (11.4%)
PE + renal vein thrombosis	2 (0.9%)
DVT + CVST	1 (0.5%)
Multiple other VTE†	3 (1.4%)
**Combined Venous + Arterial Thrombosis**	**25 (11.4%)**
CVA + DVT/PE	18 (8.2%)
Myocardial infarction + PE	2 (0.9%)
CVA + Other VTE	1 (0.5%)
Other arterial‡ + VTE	4 (1.8%)
**VTE Provoking Risk Factors**	
Provoked*	**101 (45.9%)**
Pregnancy/combined oral contraceptives	36 (16.4%)
Antecedent hospitalization	16 (7.3%)
Major surgery	14 (6.4%)
Active cancer	10 (4.5%)
Major trauma/prolonged immobilization	9 (4.1%)
Liver/gastrointestinal disease (for SVT)	8 (3.6%)
COVID-19	5 (2.3%)
Indwelling venous catheter	4 (1.8%)
Venous anomaly (May-Thurner or thoracic outlet syndrome)	4 (1.8%)
Other risk factors	7 (3.2%)
**Unprovoked**	**119 (54.1%)**

CVA, cerebrovascular accident (including stroke, retinal artery occlusion, and transient ischemic attack); CVST, cerebral venous sinus thrombosis; DVT, deep vein thrombosis; PE, pulmonary embolism

†Includes renal, ovarian, inferior vena cava, and cephalic vein thromboses

‡Includes radial artery, superficial femoral artery, left ventricular, and arteriovenous graft thromboses

*11 patients had multiple risk factors

MTHFR, methylenetetrahydrofolate reductase; N/A, not applicable; PAI-1, plasminogen activator inhibitor-1

### Test results

A total of 835 tests were ordered. The counts and percentages of each test in context of their lab outcomes are detailed in Table 2. The most commonly ordered tests were for FVL (25.1%), PT_G20210A_ (19.4%), and deficiencies of protein S (18.0%), protein C (14.5%), and antithrombin (12.8%). Overall, 19.6% of tests were abnormal, including 50.5% of antithrombin activities, 24.8% of protein C assays, 20.0% of protein S assays, and 6.7% of assays for FVL. Regarding the most common genetic tests, 9.1% of FVL and 6.2% of PT_G20210A_ tests were positive (all heterozygous, with two patients double heterozygous for FVL and PT_G20210A_). The four tests with a lower limit of normal higher than 60% were frequently borderline positive (i.e., >60% but below the normal limit), including 33.6% of antithrombin activity, 14.3% of total Protein S, 13.9% of protein C function and 7.7% of protein C antigen assays. The total institutional charges for all of the tests during the study period were $385,161, while Medicare fees totaled $26,029.55, based on CMS fee schedules for the first quarter of 2025. Table 3 displays our institution’s chargemaster rates and the CMS fee schedule for all thrombophilia tests.

**Table 2:** Results of Hereditary Thrombophilia Testing

			**Lab Results**		
**Test Condition**	No. Test(% of total)	Ref Range	Negative(% within test)	Borderline positive / Heterozygous (%)	Positive / Homozygous (%)
**All Tests**	**835 (100.0)**		**671 (80.4)**	**80 (9.6)**	**84 (10.1)**
**Factor V Leiden (FVL)**	**210 (25.1)**		**196 (93.3)**	**8 (3.8)**	**6 (2.9)**
Activated Protein C Resistance	122 (14.6)	>2.2	116 (95.1)	N/A	6 (4.9)
FVL mutation	88 (10.5)		80 (90.9)	8 (9.1)	0 (0.0)
**Prothrombin G20210A**	**162 (19.4)**		**152 (93.8)**	**10 (6.2)**	**0 (0.0)**
**Protein S Deficiency**	**150 (18.0)**		**120 (80.0)**	**4 (2.7)**	**26 (17.3)**
Protein S, free	115 (13.8)	53–141%	93 (80.9)	N/A	22 (19.1)
Protein S, total	28 (3.4)	70–140%	23 (82.1)	4 (14.3)	1 (3.6)
Protein S, activity	7 (0.8)	60–140%	4 (57.1)	N/A	3 (42.9)
**Protein C Deficiency**	**121 (14.5)**		**91 (75.2)**	**16 (13.2)**	**14 (11.6)**
Protein C, function	108 (12.9)	78–160%	81 (75.0)	15 (13.9)	12 (11.1)
Protein C, antigen	13 (1.6)	70–140%	10 (76.9)	1 (7.7)	2 (15.4)
**Antithrombin activity**	**107 (12.8)**	86–122%	**53 (49.5)**	**36 (33.6)**	**18 (16.8)**
**Hyperhomocysteinemia**	**84 (10.1)**		**59 (70.2)**	**6 (7.1)**	**19 (22.6)**
Homocysteine	75 (9.0)	5–15 µmol/L	57 (76.0)	N/A	18 (24.0)
*MTHFR* Mutation	9 (1.1)		2 (22.2)	6 (66.7)	1 (11.1)
**PAI-1 activity**	**1 (0.1)**	4.43 ng/mL	**0 (0.0)**	**N/A**	**1 (100.0)**

MTHFR, methylenetetrahydrofolate reductase; N/A, not applicable; PAI-1, plasminogen activator inhibitor-1

**Table 3:** Thrombophilia Tests with Associated Institutional Chargemaster and CMS Fees

**Thrombophilia Tests**	**TJUH Chargemaster ($)**	**CMS Fee Schedule ($)**
**Factor V Leiden (FVL)**	**- **	** - **
Activated Protein C Resistance	$334	$15.32
FVL mutation	$373	$73.37
**Prothrombin G20210A**	** $410 **	** $65.69 **
**Protein S Deficiency**	**- **	**- **
Protein S, free	$765	$15.32
Protein S, total	$765	$11.61
Protein S, activity	$268	$15.32
**Protein C Deficiency**	**- **	** - **
Protein C, function	$480	$13.84
Protein C, antigen	$480	$12.01
**Antithrombin activity**	** $480 **	** $11.85 **
**Hyperhomocysteinemia**	**- **	**- **
Homocysteine	$235	$17.92
*MTHFR* mutation	$737	$65.34
**PAI-1 activity**	** $200 **	** $17.19 **

MTHFR, methylenetetrahydrofolate reductase; PAI-1, plasminogen activator inhibitor-1

### Patient-level results

**Fig. 1:**
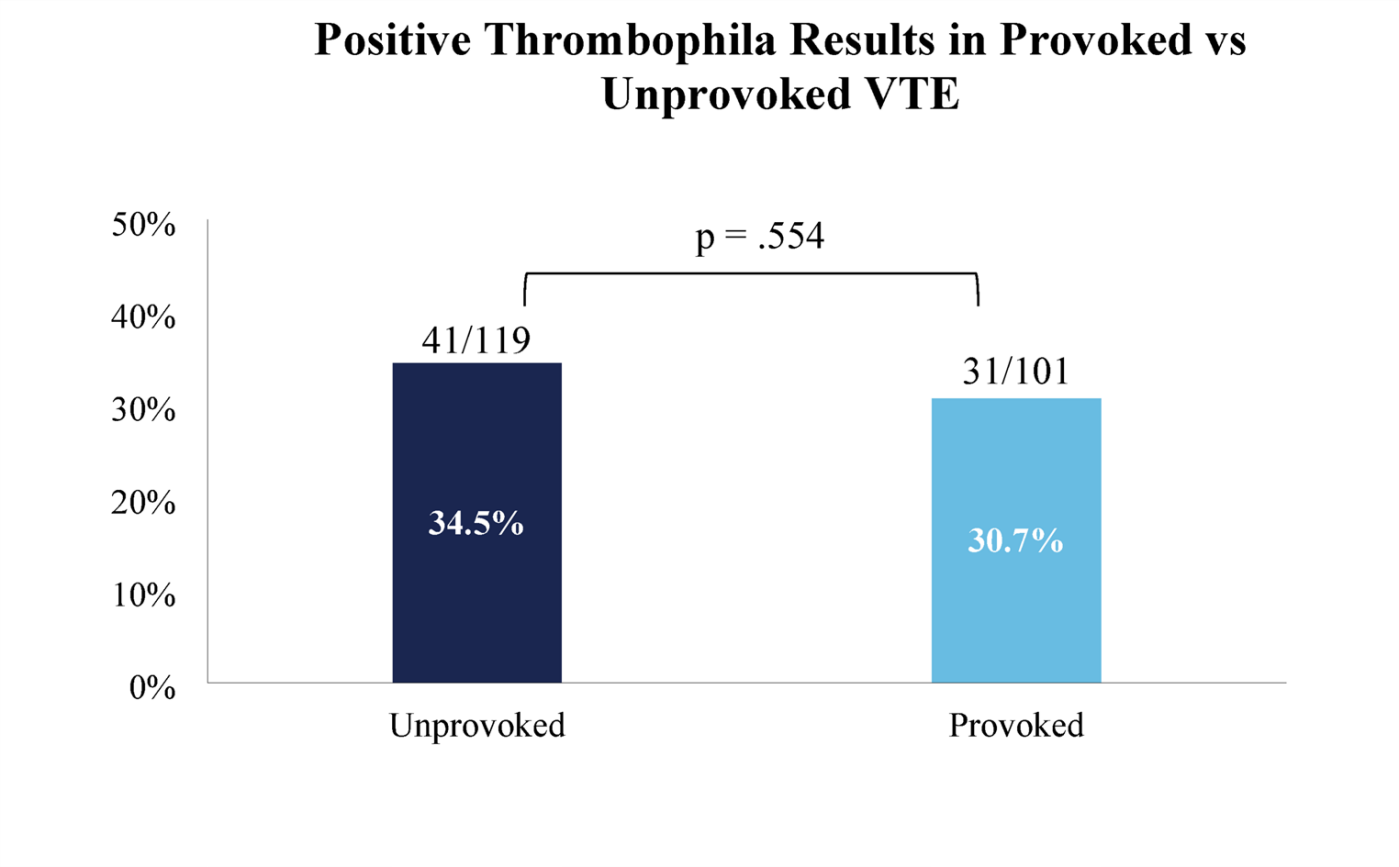
Percentage of positive lab results in patients with provoked versus unprovoked VTE

**Fig. 2:**
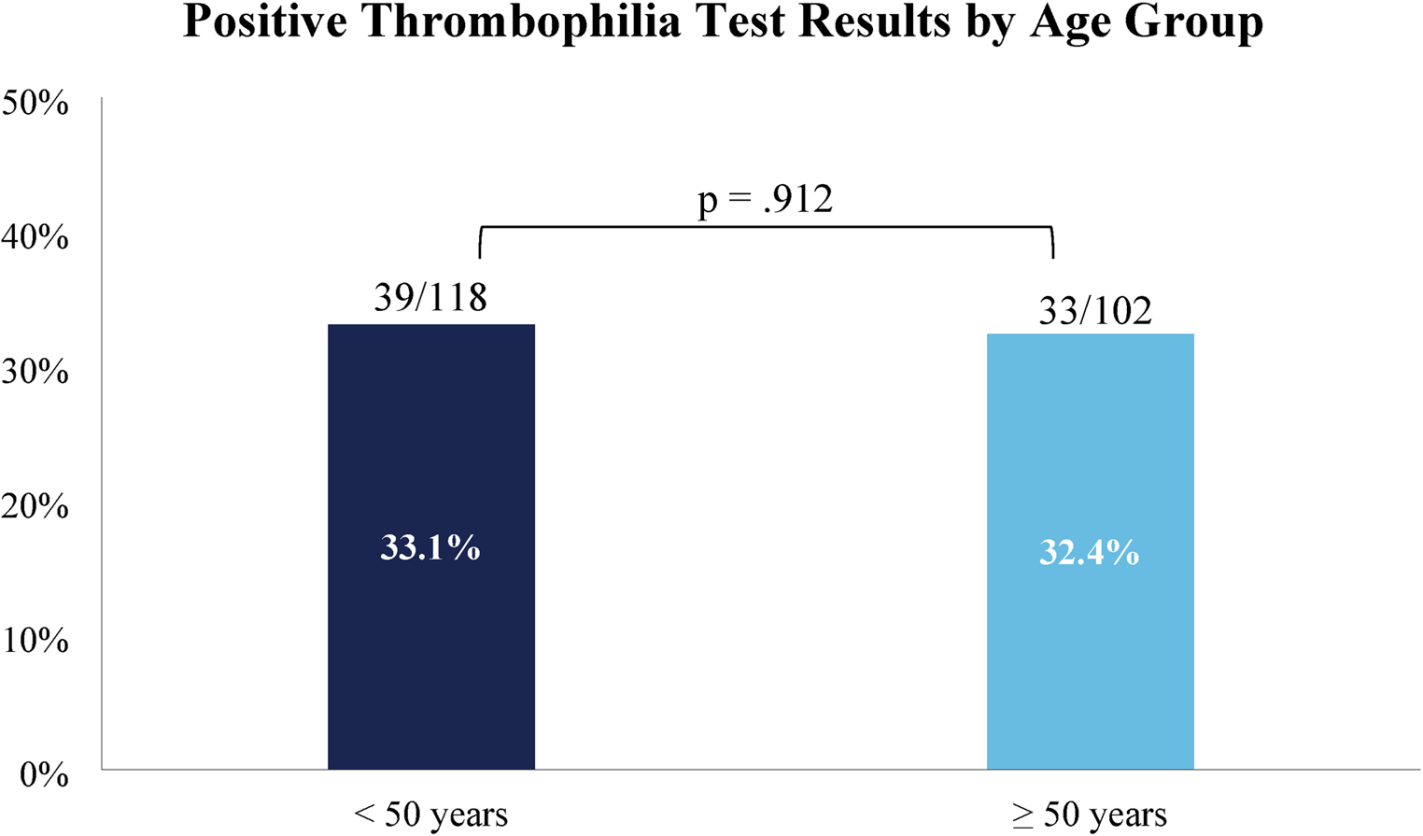
Percentage of positive lab results in patients < 50 vs. ≥ 50 years of age

We found that in the patient cohort, 99/220 (45.0%) had at least one abnormal result. Of these, 72 (32.7%) patients had a positive result on an activity assay or any gene variant, while 27 (12.3%) had only borderline positive findings. 43 (19.5%) patients had multiple abnormal results. There was no significant difference in the rate of positive test results among patients whose thromboses were provoked vs. unprovoked (69.3% vs. 65.5%, *p* =.554) (Fig. 1). We also observed no association between age and likelihood of a positive result. 33.1% of patients age < 50 years had a positive result vs. 32.4% of patients ≥ 50 years (*p* =.912) (Fig. 2), and the mean age of patients with positive results was similar (47.6 vs. 48.3 years, *p* =.794). When evaluating test results based on the type of presentation, the rate of positive tests was similar among patients with VTE only vs. combined VTE and arterial thrombosis (31.8% vs. 40.0%, *p* =.410). Finally, the study includes 32 (14.5%) patients whose VTE at the time of thrombophilia testing represented a recurrence, defined as an imaging-proven thrombosis in a different location as the initial event(s) or following documented resolution [25]. We also considered patients with clear progression of VTE despite standard-of-care anticoagulant treatment as having a recurrence. There was no significant difference in mean age of patients with first or recurrent VTE (47.6 vs. 51.0 years, *P* =.299), nor in the rate of unprovoked VTE (53.2% for first VTE vs. 59.4% for recurrent, *P* =.325). Regarding test results, patients presenting with first vs. recurrent VTE were similarly likely to have a positive thrombophilia test result (34.6% vs. 21.9%, *P* =.221).

 Regarding management, at a median time to last documentation of 215 days, only 4 out of 99 (4.0%) patients with any abnormal result had a definite, documented change in management that was implemented after the abnormal result became available – either adding an anticoagulant, changing it, or prolonging the duration. An additional 6 (6.1%) patients had results which may have influenced management, but for whom documentation was inconclusive in demonstrating a link between clinical management and abnormal results. The characteristics of the four patients with a clear change in management in response to an abnormal test are summarized in Table 4. They included patients with a provoked DVT with heterozygosity for both FVL and PT_G20210A_, an unprovoked CVST heterozygous for PT_G20210A_, a hormone-associated PE with low free protein S, and an unprovoked CVST heterozygous for both FVL and PT_G20210A_. In 3 of the 4 cases, the indication for testing fit the 2023 ASH recommendations. We observed no difference in the number of patients whose management clearly changed according to whether the VTE was provoked vs. unprovoked (4.1% vs. 4.0%). Among the 84 patients with an abnormal result(s) on an antigen or functional assay, only 8 (9.5%) had the abnormal test(s) repeated. Finally, 6 (2.7%) patients died during the index hospitalization.

**Table 4:** Characteristics of patients with documented management change following positive result

Clinical Scenario	Provoking Risk Factor(s)	Abnormal Test Result(s)	Documented Clinical Decision Change	Compatible with 2023 ASH Guidelines
33-year-old male with distal (gastrocnemius and peroneal veins) DVT	Yes: traumatic spinal cord injury and surgery within 14 days	Heterozygous for both FVL and PT_G20210A_	Prescribed long-term apixaban at dose of 2.5 mg BID due to thrombophilia and permanent immobility	No
56-year-old male with CVST	None	Heterozygous for PT_G20210A_	Discharged on warfarin, switched to apixaban 2.5 mg BID as long-term therapy	Yes
20-year-old female with massive PE requiring thrombolysis	Yes: Combined oral contraceptive	Low free protein S (26%), normal total protein S (70%)	Prescribed long-term rivaroxaban 10 mg due in part to free protein S result	Yes
22-year-old male with CVST	None	Heterozygous for both FVL and PT_G20210A_	Maintained on rivaroxaban 20 mg as long-term therapy	Yes

## Discussion

In this 5-year retrospective study of inpatient hereditary thrombophilia testing after acute VTE, we found that abnormal results were very common and rarely changed clinical management. These findings highlight a disconnect between current practice and most evidence-based recommendations. Testing was frequently performed immediately following a VTE event, when results are more likely to be misleading, and was associated with significant costs.

First, our study found a high rate of test utilization in groups for whom hereditary thrombophilia testing has not been recommended. British Society for Haematology guidelines advise against routine thrombophilia testing when results are unlikely to affect clinical management decisions [21]. These further note that the presence of hereditary thrombophilias rarely alters intensity, choice, or monitoring of anticoagulant therapy, and instead emphasize using clinical history and assays such as D-dimer in selected patients to guide management [21]. Similarly, ASH Choosing Wisely^®^ recommendations include not ordering thrombophilia tests after VTE by a major risk factor [20]. However, multiple studies have reported high rates of thrombophilia testing after a provoked VTE [13–15, 26–29]. Our study similarly found testing following provoked VTE was very common, comprising 45.9% of our patients and 47.3% of total tests. Even excluding VTE provoked by pregnancy or estrogen – a group for whom recent ASH guidelines support testing [22] – 29.5% of patients had a provoked VTE. Furthermore, while some advocate for selective thrombophilia testing in young patients and those without a strong provoking risk factor [30], we found that the rate of positive tests was not significantly greater in patients with unprovoked as compared to provoked VTE, nor in patients younger than 50 years of age or those with recurrent VTE.

The utility of thrombophilia testing is further limited by the potential for false positives when ordered in the acute setting, which our study confirmed. Testing following acute VTE is more likely to yield false positive results due to anticoagulation therapy that affects certain functional assays, protein consumption by acute thrombus or mechanical circulatory support systems, or acute phase reactant changes related to inflammation and/or the predisposing condition to VTE [10, 11, 26, 31]. We found 21.9% of antigen and functional assays with a lower limit of normal of 70% or higher yielded results below normal but above typical values in autosomal dominant deficiency, indicating a high likelihood of being a false positive. This has the potential to yield mismanagement of patients when results are misinterpreted or not repeated.

Furthermore, we found that fewer than 10% of patients with an abnormal antigen or activity assay had repeat testing for confirmation after the hospitalization. Others have found that while abnormal tests may be repeated, it is often during the index admission, when false positive results are still likely [15]. By focusing solely on inpatient testing after an acute VTE event, our study adds to the current literature by highlighting the importance of timing in performing hereditary thrombophilia testing. Given the high rate of likely false abnormal results and low rate of test repetition for confirmation, our data suggests that clinicians should defer testing to after an acute VTE and that guidelines such as those from ASH should directly address timing of testing in order to reduce confounding variables and the risk of clinical mismanagement of patients.

The management of patients in our study reflects that clinicians likely viewed the test results with skepticism. Only 4% of patients with any abnormal result had a documented change in clinical management in response to an abnormal result. This is consistent with other studies which found that inpatient thrombophilia testing was not clinically useful or reliable and rarely changed management [13, 14, 27, 28]. Additionally, many of these studies included acquired thrombophilias such as antiphospholipid syndrome, which often requires a change in anticoagulant – specifically to warfarin – and thus may be a higher yield test to order, especially in patients for a whom a high clinical suspicion exists. One single-center study, for example, found that 87% of patients were tested inappropriately and in no cases was the duration of treatment impacted by the result. However, among the 7 patients who experienced any clinical change in management, all had antiphospholipid syndrome, which was not included in our study [14]. Together with our study, this reinforces the concept that these tests rarely provide valuable data that impacts treatment decisions.

Finally, thrombophilia testing is expensive. When these tests are used inappropriately, they offer minimum clinical benefit but also contribute to significant spending, which may be costly to health care systems when ordered in the inpatient setting [13, 15, 26, 29]. Although cost-analysis of thrombophilia testing in acute VTE is sparce, estimates for the panel of tests included in our study range from approximately $187 to $974 USD per patient [14, 15, 26, 28, 32]. To provide a broad estimate of costs, we calculated charges using both our institution’s chargemaster and the most recent publicly available CMS fee schedule. In our study, the total institutional charges by chargemaster were greater than $385,000 USD and by CMS fee schedule greater than $26,000 USD over the 5-year study period. The corresponding per-patient charges were $1,750 and $118 USD, respectively. The implication of our findings is that reducing the utilization of these tests – or limiting them to guideline-recommended situations – represents a potential cost savings opportunity for the US health care system.

Our study has certain limitations. The retrospective, observational design from a single academic hospital system may limit the applicability of our findings to other institutions or patient populations. Additionally, the reliance on reviewing documentation to understand clinical outcomes and indications for therapy represents a weakness. Incomplete documentation, particularly from outside our EHR system, restricted our ability to fully understand and assess outcomes and potential changes in management. Many patients had no documented encounters after hospital discharge, which limited our ability to assess long-term clinical management, but also highlights the low yield of thrombophilia testing when ordered inpatient. To mitigate challenges due to incomplete documentation, charts were reviewed independently by both members of our study team, and the median time from testing to the last VTE-related documentation was 215 days, indicating extensive follow-up for many patients. Another limitation is that our study did not specifically measure the percentage of patients who were receiving anticoagulation – and which agent was being used – at the time of thrombophilia testing, which would have provided insight into reasons for false positive results. It is noted, however, that most patients had been started on anticoagulation before testing was ordered. Important strengths of this study include its use of real-world data and long study period and follow-up time, which can add valuable insights into clinical practice around thrombosis care – especially in the acute care setting – and we feel may inform future guidelines, especially those addressing the timing of testing.

## Conclusion

Our retrospective study of inpatient hereditary thrombophilia testing at an academic hospital system revealed a high rate of testing in patients with acute VTE, despite many clinical guidelines discouraging to do so. We found that in the presence of abnormal thrombophilia test results, clinical management rarely changed, likely due in part to high rates of presumed false positive results and utilizing the tests inappropriately, such that no result would alter management. We also found high costs associated with these tests. This study sheds light on the disconnect between clinical practice and evidence-based recommendations regarding hereditary thrombophilia testing. We feel it provides further evidence supporting a more selective approach to ordering thrombophilia tests in clinical practice and a shift away from the acute, inpatient setting when testing is utilized.

## Data Availability

No datasets were generated or analysed during the current study.
